# Proteome and phosphoproteome analysis of 2,4-epibrassinolide-mediated cold stress response in cucumber seedlings

**DOI:** 10.3389/fpls.2023.1104036

**Published:** 2023-02-21

**Authors:** Mengdi Zhou, Yansu Li, Yan Yan, Lihong Gao, Chaoxing He, Jun Wang, Quan Yuan, Li Miao, Shuzhen Li, Qinghua Di, Xianchang Yu, Mintao Sun

**Affiliations:** ^1^ State Key Laboratory of Vegetable Biobreeding, Institute of Vegetables and Flowers, Chinese Academy of Agricultural Sciences, Beijing, China; ^2^ Beijing Key Laboratory of Growth and Developmental Regulation for Protected Vegetable Tablecrops, China Agricultural University, Beijing, China; ^3^ College of Horticulture, Sichuan Agricultural University, Chengdu, China; ^4^ College of Horticulture, Zhejiang A & F University, Hangzhou, China; ^5^ College of Life Science, Gannan Normal University, Ganzhou, China

**Keywords:** brassinosteroid (BR), cold stress, cucumber seedling, phosphoproteome, proteome

## Abstract

The 2, 4-epibrassinolide (EBR) significantly increased plants cold tolerance. However, mechanisms of EBR in regulating cold tolerance in phosphoproteome and proteome levels have not been reported. The mechanism of EBR regulating cold response in cucumber was studied by multiple omics analysis. In this study, phosphoproteome analysis showed that cucumber responded to cold stress through multi-site serine phosphorylation, while EBR further upregulated single-site phosphorylation for most of cold-responsive phosphoproteins. Association analysis of the proteome and phosphoproteome revealed that EBR reprogrammed proteins in response to cold stress by negatively regulating protein phosphorylation and protein content, and phosphorylation negatively regulated protein content in cucumber. Further functional enrichment analysis of proteome and phosphoproteome showed that cucumber mainly upregulated phosphoproteins related to spliceosome, nucleotide binding and photosynthetic pathways in response to cold stress. However, different from the EBR regulation in omics level, hypergeometric analysis showed that EBR further upregulated 16 cold-up-responsive phosphoproteins participated photosynthetic and nucleotide binding pathways in response to cold stress, suggested their important function in cold tolerance. Analysis of cold-responsive transcription factors (TFs) by correlation between proteome and phosphoproteome showed that cucumber regulated eight class TFs may through protein phosphorylation under cold stress. Further combined with cold-related transcriptome found that cucumber phosphorylated eight class TFs, and mainly through targeting major hormone signal genes by bZIP TFs in response to cold stress, while EBR further increased these bZIP TFs (CsABI5.2 and CsABI5.5) phosphorylation level. In conclusion, the EBR mediated schematic of molecule response mechanisms in cucumber under cold stress was proposed.

## 1 Introduction

Cold stress was the primary environmental stress affecting plant growth and development (Zhang et al., 2016; Zaynab et al., 2017; Guo et al., 2019), which resulted in plant chlorosis, growth retardation, and even death (Xia et al., 2018; Garzon et al., 2020). Thus, plants had thus developed sophisticated cellular and molecular machinery to cope with cold stress (Sergeant et al., 2014), eventually leading to various biological and physiological responses, including stomatal closure, inhibiting cell growth, and changing photosynthesis and respiration (Khan et al., 2017; Khan et al., 2019).

The physiological and biochemical metabolism, such as plant proteins, amino acids, and signal transduction changed considerably under cold stress (Wang et al., 2016). So far, an extensive array of stress-responsive genes had been identified in plants, including-cold regulated (*COR*), cold-induced (*KIN*), low-temperature-induced (*LTI*), and responsive to dehydration (*RD*) (Tao et al., 2014; Chilundo et al., 2017; Kindgren et al., 2018). Recently, most studies were mainly focused on transcription levels (Ning et al., 2019; Tian et al., 2022; Zheng et al., 2022). It is well known that mRNA is an essential tool for protein production, and proteins ultimately carried out their functions in cells (Kosová et al., 2018). Therefore, it is important to study the proteome’s changes to understand the plant’s response during abiotic stress. With the development of proteomics sequencing, proteomics related to cold stress has been studied in plants such as *Arabidopsis*, tomato, rice, and other plant species (Hsu et al., 2018; Sant’Ana and Lefsrud, 2018; Trentmann et al., 2020). Moreover, studies found that certain differentially expressed mRNAs did not cause protein differences, which may be due to the effect of post-transcriptional modification (PTM) regulatory mechanisms on protein content (Wang et al., 2018a; Jin et al., 2019; Rodon et al., 2019). About 300 types of experimentally verified or putative PTMs have been reported (Zhong et al., 2017), mainly including glycosylation, acetylation, and phosphorylation, among which phosphorylation is one of the most important, common, and well-studied PTMs in proteins (Hou et al., 2015). Protein phosphorylation is a reversible covalent binding. The phosphate group binds to the hydroxyl groups of hydroxyl amino acids such as serine, threonine, and tyrosine, but occasionally to the hydroxyproline (Reinders and Sickmann, 2010). In contrast, dephosphorylation refers to the reversible removal of phosphate groups from proteins. The protein phosphorylation/dephosphorylation participated in the regulation of protein stability and activity, affecting signal transduction, which finally changed plants’ physiological and biochemical metabolism (Tichy et al., 2011; Chen and Weckwerth, 2020). Recently, the high-throughput sequence of proteome and phosphoproteome for plants under cold stress had been only reported in tomatoes, bananas, and *Arabidopsis* (Sanchez-Bel et al., 2012; Yang et al., 2012; Nakaminami et al., 2014; Gao et al., 2017; Hsu et al., 2019; Kamal et al., 2020). However, multiple omics analyses of the transcriptome, proteome, and phosphorylation for plants under cold stress have still not been reported.

Hormone signaling plays a crucial role in plant tolerance to abiotic stress (Wang et al., 2019a). Brassinosteroid (BR) is one of the most important phytohormones and plays a central role in cell division, senescence, and photosynthesis (Khan et al., 2017; Khan et al., 2019). Furthermore, BRs also improved plant tolerance to abiotic stress (Khan et al., 2017), such as cold (Xia et al., 2018), salt (Li et al., 2021), drought (Wang et al., 2019b), and others (Kohli et al., 2018; Zhou et al., 2018). Studies have revealed that BR is essential in integrating signals and regulating the expression of cold-responsive genes. It can increase the expression of cold-responsive genes and regulate multiple signaling pathways to increase cold tolerance in plants (Eremina et al., 2016a; Li et al., 2017; Ye et al., 2019). BR was perceived by the cell-surface receptor kinases BR-insensitive 1 (*BRI1*) to begin BR signal transduction. BR signals are transmitted in two main ways in *BRI1* downstream, including phosphorylation and dephosphorylation of *BIN2* (Guo et al., 2013; Wang et al., 2014) and the *BZR/BES* binding target gene promoter (Guo et al., 2013). Therefore, the 2,4-epibrassinolide (EBR) was used for improving the cold tolerance of many plants (Xia et al., 2014; Anwar et al., 2018; Bai et al., 2018; Dou et al., 2021). However, in plants, how EBR regulates phosphoproteome and the combined analysis of EBR regulation of phosphoproteome and proteome under cold stress have not been reported.

Cucumber is one of the most important economic crops globally, which is sensitive to cold stress. Cold stress is one of the major environmental stresses that adversely affects the protected cultivation of cucumber for its growth and yield. Therefore, studying the regulation mechanism of cucumber response to cold stress at the multi-omics level is crucial. Here, for the first systematic analysis, EBR-regulated cold stress response in multi-omics levels and EBR-mediated schematic of molecule response mechanisms in cucumber under cold stress were proposed.

## 2 Materials and methods

### 2.1 Preparation of 2,4-epibrassinolide

The 2,4-epibrassinolide (EBR, Sigma-Aldrich Corporation, St. Louis, MO, USA) stock solution (20 mg/ml) was prepared by dissolving the required quantity in 1.14 ml of ethanol, and the final volume was maintained up to 500 ml with distilled water.

### 2.2 Plant material and growth conditions

The cucumber seedlings were grown in an artificial chamber at the Institute of Vegetables and Flowers, Chinese Academy of Agricultural Sciences, Beijing, China. Cucumber seeds of the “Changchunmici” cultivar were provided by the facility cultivation research group of CAAS. Cucumber seeds were soaked for 5 h in water at 55°C. The seeds were germinated for 2 days under dark conditions at 28°C and then sown in the bowl (9 cm × 9 cm) filled with a growth medium composed of grass charcoal and vermiculite (volume ratio 2:1). We grew seedlings at conventional growth conditions (25°C day/18°C night, 16 h of light) photoperiods.

### 2.3 Treatments and sampling

Seedlings with similar growth patterns were divided into three groups at the two-leaf stage (25–30 days): CK, 25°C/18°C, Cold (Cold, 4°C), and EBR-Cold (treated with 0.1 mg/ml EBR, 4°C). Before cold treatment, the CK and Cold groups were foliar sprayed with the same concentration of ethanol (containing 0.01% Tween 20). The Cold and EBR-Cold groups suffered chilling stress at 8 am the next day after spraying, and CK groups at normal temperature (25°C/18°C, day/night). After 4 h of chilling treatment, the cucumber leaves (the second leaves from the bottom) were sampled and then stored at −80°C. Each treatment had three biological replicates (nine pots per replicate).

When cucumber seedlings’ growth patterns reached the two-leaf stage, seedlings with consistent growth were treated at 4°C. At 0 and 36 h post-treatment, cucumber leaves were collected and then stored at −80°C. Each treatment had three biological replicates (nine pots per replicate) for qRT-PCR. Proteome and phosphoproteome were conducted with two biological replicates (nine pots per replicate), respectively.

### 2.4 Real-time PCR

Total RNA was extracted with an RNA extraction kit (Huayue Biotechnology, Huayue Yang, Beijing, China), and first-strand cDNA was synthesized using Fast Quant RT Kit (Huayue Biotechnology, Huayue Yang, Beijing, China). PCR was then carried out using the gene-specific primers listed in **Supplementary Table S11** and the Super Real Premix Plus (SYBR Green) Kit (Mei5 Biotechnology, Co., Ltd., Beijing, China) with an Mx3000p Real-time PCR System (Stratagene, Agilent Technologies, Inc., Santa Clara, CA, USA). We used the 2^−ΔΔCt^ methods to calculate gene expressions (reference gene: *CsActin*), and then SPSS software was used to analyze the significance of differences using Tukey’s HSD (*p* < 0.05). Each sample had three biological replicates.

### 2.5 Determination of malondialdehyde and chilling injury index

The MDA was determined by the thiobarbituric acid (TBA) colorimetric method (Jiang and Zhang, 2001).

Referring to the methods of Yu (1998), chilling injury symptoms in seedlings can be graded for five levels. According to this method of calculation, the cold injury index is. The calculation formula is as follows:

Chilling Injury Index = (1 × R1 + 2 × R2 + 3 × R3 + 4 × R4 + 5 × R5 + 0 × R0)/(Number of plants per treatment × 5);

R0–R5 is the number of seedlings of grades 0–5, respectively.

### 2.6 Quantitative proteome analysis

#### 2.6.1 Protein extraction

All experiments were performed in biological triplicates for different treatments (CK, Cold, and EBR-Cold), and sequencing library construction was performed independently. Libraries from triplicate samples were combined and sequenced, and we repeated two independent experiments. Four volumes of Tris-saturated phenol (1% of a cocktail of protease and phosphatase inhibitors, 10 mM dithiothreitol) had been added to the sample powder. The Tris-saturated phenol was added after facilitating lysis by sonication. The supernatants were collected after centrifugation, and proteins were precipitated with ammonium acetate in methanol overnight at −20°C. Precipitated products were washed with methanol and acetone after being separated by filtration. Finally, the precipitate was treated with 8 M urea, and a BCA kit measured the protein concentration.

#### 2.6.2 Protein digestion

Equal amounts of total proteins from CK, EBR, and EBR-Cold were taken to perform enzymatic hydrolysis. The volume of protein was adjusted to 400 μl with lysis buffer, adding appropriate volumes of standard proteins. Trichloroacetic acid was slowly added and mixed well by vertexing for 2 h at 4°C. The precipitate was then centrifuged (4,500×*g*, 5 min, 4°C) and washed with precooled acetone before air drying. The precipitates were resuspended in tetraethylammonium bromide (TEAB, 200 Mm) and then disrupted by sonication. Trypsin was added to further digest proteins overnight (15 h) at room temperature followed by DDT, which was reduced it for 30 min at 56°C. Iodoacetamide at 11 mM was added immediately and allowed to sit at room temperature for 15 min.

#### 2.6.3 TMT labeling and HPLC fractionation

After trypsin digestion, the peptides were desalted (Strata XC18 SPE column) and vacuum dried. TEAB was used to mark the peptides, and a TMT kit was used to treat them. After 2 h at room temperature, the peptide mixtures were pooled, desalted, and vacuum centrifuged for drying. After being labeled, the sample was then fractionated into fractions by high pH reverse-phase HPLC (Agilent 300 Extend C18) by Wang et al. (2018).

#### 2.6.4 LC-MS/MS analysis

The peptides were subjected to an NSI source followed by tandem mass spectrometry (MS/MS) in Q Exactive TM Plus (Thermo, USA) coupled online to the UPLC. Eluted peptides were separated by UHPLC reverse phase chromatography (EASY-nLC 1000) (Wang et al., 2020). Next, the peptides were separated and ionization through the ultra-high-performance liquid phase system and NSI ion source, respectively, and intact peptides were detected in the Orbitrap Lumos™. Peptides were then selected for MS/MS using NCE, and high-resolution Orbitrap was used to detect and analyze peptide precursor ions and their secondary fragments. Data-dependent acquisition (DDA) was used for data acquisition.

#### 2.6.5 Database search

The resulting MS/MS data were processed using the Maxquant search engine (v.1.5.2.8). Tandem mass spectra were searched against the UniProt database concatenated with the reverse decoy database. Trypsin/P was specified as a cleavage enzyme, allowing up to four missing cleavages. The mass tolerance for precursor ions was set as 20 ppm in the first search and 5 ppm in the main search, and the mass tolerance for fragment ions was set as 0.02 Da. Carbamidomethyl on Cys was specified as a fixed modification, and acetylation modification and oxidation on Met were specified as variable modifications. FDR was adjusted to < 1%, and the minimum score for modified peptides was set to > 40.

#### 2.6.6 Protein annotation

Secondary mass spectral data were processed using the Maxquant software v1.5.2.28. The database information is Blast_Cucumis_sativus_3659_PR_20191030.fasta (23,744). The false-positive rate (FDR) was adjusted to < 1%. The database was downloaded from the UniProt website (https://www.uniprot.org, creation date: 30 October 2019). The decoy database was generated by reversing all target protein sequences from the target database. The number of the decoy database is the same as that of the target database. The retrieval parameters were set as follows: the database was Cucumis_sativus_3659 (23,744 sequences), an antidatabase is added to calculate the FDR caused by random matching, and a common contamination library was added to the database to eliminate the contamination of the protein in the identification results. Cleavage specificity set to trypsin/P, with two missed cleavages allowed.

### 2.7 Phosphoproteome sequence and analysis

#### 2.7.1 Phosphoprotein extraction

All experiments were performed in biological triplicates for different treatments (CK, Cold, and EBR-Cold), and sequencing libraries were constructed independently. Libraries from triplicate samples were combined and sequenced in two separate experiments. The lysis buffer of phosphoprotein extraction contained phosphatase inhibitors (PhosSTOP, Roch, Germany), and the extraction method was the same as in Protein extraction.

#### 2.7.2 Trypsin digestion

The trypsin digestion of the phosphoproteome sequence is discussed in the Protein digestion section.

#### 2.7.3 Phosphopeptide enrichment

The peptides were dissolved in a loading buffer containing 2% trifluoroacetic acid (TFA) and 60% acetonitrile (ACN) saturated glutamine solution. A fourfold volume of titanium dioxide suspension was added to collect the sediment. The sediment was washed twice with wash buffer 1 (0.5% TFA and 50% ACN) and wash buffer 2 (0.1% TFA and 50% ACN). The peptides are then washed off with 10% NH_3_·H_2_O. Following the preceding steps, the supernatant contained the final enriched phosphopeptides solution, which was vacuum concentrated for further use.

#### 2.7.4 TMT labeling and HPLC fractionation

The TMT labeling and HPLC fractionation of the phosphoproteome sequence are discussed in the TMT labeling and HPLC fractionation section.

#### 2.7.5 Mass spectrometry

The mass spectrometry of the phosphoproteome sequence is discussed in the LC-MS/MS analysis section.

#### 2.7.6 Phosphopeptide annotation

The database search of the phosphoproteome sequence is discussed in the Database search section.

### 2.8 Data analysis

The cucumber transcriptome dataset (GSE111998) was used in this study. Screening criteria for differential genes include the following: fold change ≥ 2 and *Q* value < 0.05. Screening criteria for differential proteins and phosphopeptides include the following: fold change ≥ 1.2 and *Q* value < 0.05. All differential phosphopeptides for a protein are up- or downregulated and are called “same pattern” peptides, and phosphopeptides with opposite regulation for a protein are called “different pattern” peptides. We found these two types of peptides using a Perl script.

The command used for protein blast analysis is as follows: blastall -i input.fa -d data_base.fa -p blastp -e 1e-10 -b 1 -v 1 -m 8 -o blast.out. The above command obtained homologous proteins between cucumber and other plant species.

GO annotations of proteome and phosphoproteome data were performed based on the *UniProt-GOA* database (http://www.ebi.ac.uk/GOA/). KEGG pathway annotation analysis was performed utilizing the KEGG pathway database (http://www.genome.jp/kegg). The differential phosphopeptides and proteins were analyzed for their enrichment on GO and KEGG, then tested by Fisher’s exact test (*p* < 0.05). The String 11.0 software was used to construct a high-quality protein interaction network for these differential genes with a score greater than 700. The Cytoscape software was then used to construct the protein–protein interaction (PPI) network.

To predict conserved phosphorylation motifs, peptides of 15 amino acids in length with phosphorylated residues in the center were searched against all the identified proteins in the Motif-X online search engine (https://motif-x.med.harvard.edu/) (Schwartz and Gygi, 2005). The occurrence threshold was set at 20, and the *p*-value threshold was set at < 10^−6^.

## 3 Results

### 3.1 The 0.1 mg/ml EBR improved the cold tolerance of cucumber seedlings

Pretreated cucumber seedlings with 0.1 mg/ml EBR significantly decreased the chilling injury index and enhanced cold tolerance (**Supplementary Table S1**; **Supplementary Figure S1**). The cold treatment caused an increase in *CsICE1* and *CsCBF1*, *CsCBF12*, and *CsCBF13* expressions compared with CK; EBR-Cold treatment caused a significant increase in all *CsICE* and *CsCBF* expressions compared with CK (**Supplementary Figure S2A**). Compared with CK, cold treatment significantly decreased the expression of BR biosynthetic genes (*Cs90A1* and *Cs90B1*), and EBR-Cold treatment further increased the two genes’ expression (**Supplementary Figure S2B**). Additionally, cold treatment significantly increased the expression of *CsBES2*, *CsBES3*, *CsBIN1*, *CsBIN2*, and *CsBRI1*, and EBR-Cold treatment significantly increased the expression of *CsBES1*, *CsBES2*, and *CsBES3* (**Supplementary Figure S2C**). Therefore, 0.1 mg/ml EBR enhanced cold tolerance and could be used for further phosphoproteome and proteome sequencing.

### 3.2 Effects of EBR in phosphoproteome and proteome

**Figure 1:**
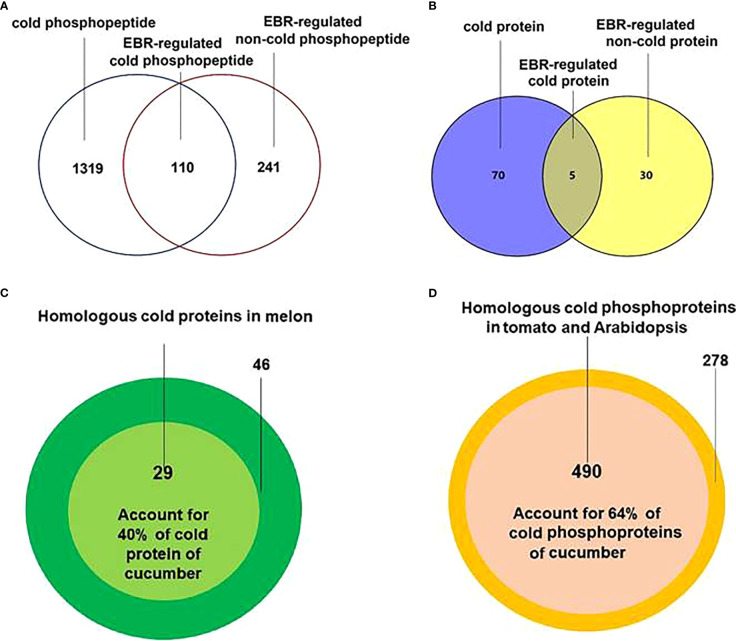
Extraction and alignment of differential proteins and differentially expressed phosphoproteins. **(A)** The Venn of differentially expressed proteins in different treatments. **(B)** The Venn diagram of differentially expressed phosphoproteins in different treatments. **(C)** Cold-responsive protein alignment between cucumber and melon. **(D)** Cold-responsive phosphopeptide corresponding protein alignment between cucumber and *Arabidopsis* and tomato.

The differential phosphopeptides (**Figure 1A**; **Supplementary Figure S3A**; **Supplementary Table S2**) and proteins (**Figure 1B**; **Supplementary Figure S3B**; **Supplementary Table S3**), including cold-responsive, EBR-regulated cold-responsive, and EBR-regulated non-cold-responsive, were then extracted by using DESeq (Anders and Huber, 2010). Further protein sequence alignment analysis found that there were 29 cold-responsive proteins in melon (Song et al., 2020) (**Figure 1C**; **Supplementary Table S4**) and 490 cold-responsive phosphoproteins in *Arabidopsis* and tomato (Sanchez-Bel et al., 2012; Kamal et al., 2020) (**Figure 1D**; **Supplementary Table S5**) that were highly homologous to cold-responsive proteins and phosphoproteins in cucumber, accounting for 40% and 64% of cold-responsive proteins and phosphoproteins in cucumber, respectively. These results indicated that the cold-responsive proteins and phosphoproteins were likely to be conserved in some plants and further demonstrated the accuracy of sequencing in cucumber seedlings.

**Figure 2:**
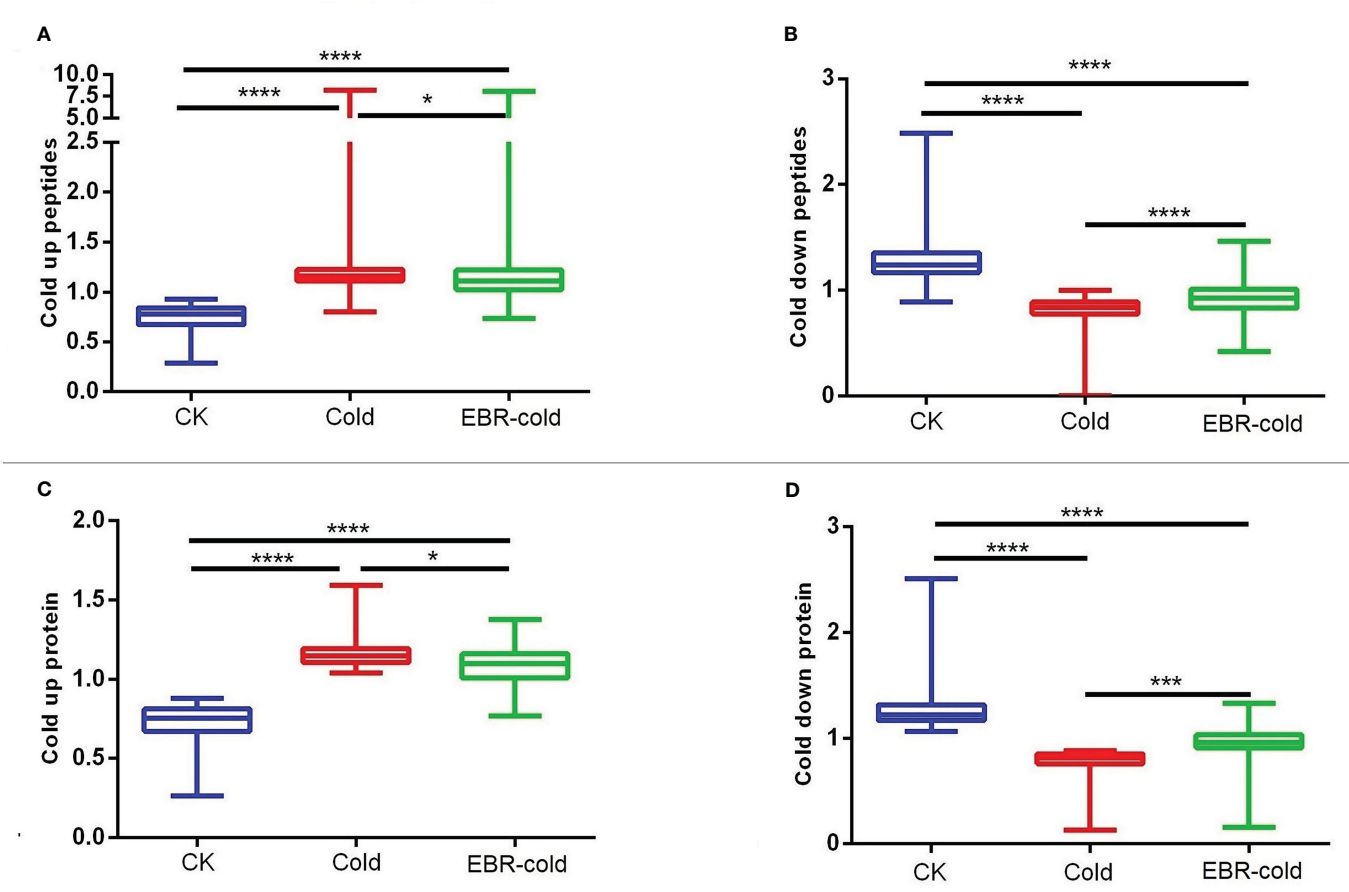
The regulation of EBR in cold-responsive phosphopeptides and cold-responsive proteins. **(A, B)** Cold-responsive phosphopeptides; **(C, D)** cold-responsive proteins. The "*" indicates significantly different (p < 0.05 by Tukey’s test), the "***" indicates significantly different (p < 0.001 by Tukey’s test), and "****" indicates significantly different (p < 0.0001 by Tukey’s test).

We further studied the effect of EBR on the regulation of cold-responsive phosphopeptides or proteins. We found that EBR downregulated cold-up phosphopeptides (**Figure 2A**) but upregulated cold-down phosphopeptides (**Figure 2B**). Similarly, EBR downregulated cold-up proteins (**Figure 2C**) but upregulated cold-down proteins (**Figure 2D**). Hence, EBR negatively regulated the phosphorylation level of cold-responsive phosphopeptides and cold-responsive protein content.

### 3.3 The phosphorylation site analysis under cold stress of cucumber seedlings

**Figure 3:**
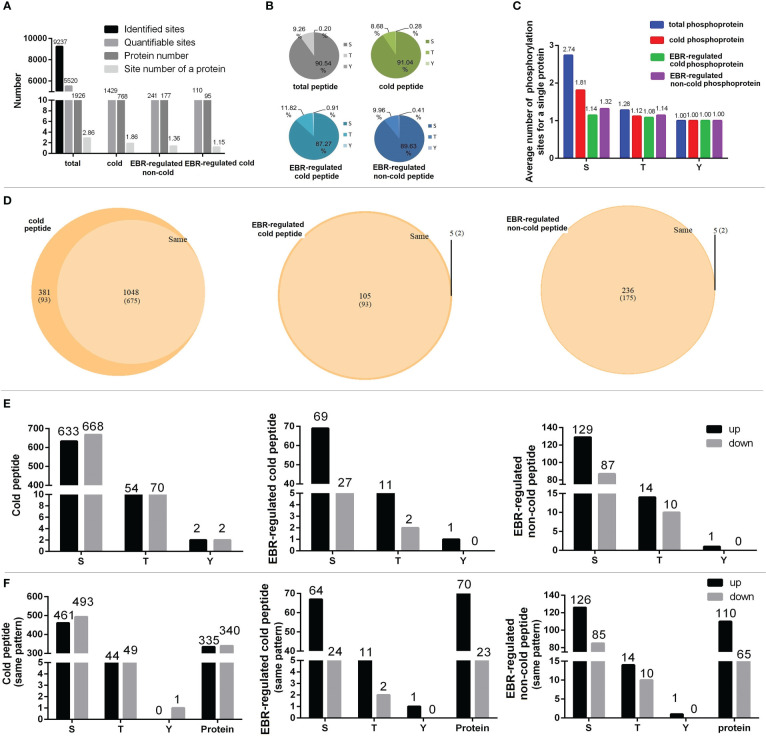
Analyses of phosphorylation patterns in cold-responsive phosphopeptides, EBR-regulated cold-responsive phosphopeptides, and EBR-regulated non-cold-responsive phosphopeptides. **(A)** The phosphorylation site statistics; **(B)** proportional of Ser, Thy, and Tyr sites in differential phosphoproteins; **(C)** an average number of phosphorylation sites for a single protein; **(D)** proportional of “same-pattern” and “different-pattern” peptides in phosphoproteome; **(E)** the number of up- or downregulated differential Ser, Thr, and Tyr phosphorylation sites in the differential phosphopeptides; and **(F)** the number of up- or downregulated differential Ser, Thr, and Tyr phosphorylation sites in the differential phosphoproteins.

We detected 9,237 phosphorylation sites in the phosphoproteome, of which 5,520 phosphorylation sites on 1,926 proteins presented quantitative information, with an average of three phosphorylation sites per protein. There are 1,429 cold-responsive phosphorylation sites on 768 proteins, an average of nearly two phosphorylation sites per protein. There are 241 EBR-regulated non-cold-responsive phosphorylation sites on 177 proteins, with an average of one phosphorylation site per protein, and 110 EBR-regulated cold-responsive phosphorylation sites on 95 proteins, with an average of one phosphorylation site per protein. Therefore, cold stress induced multi-site phosphorylation but EBR induced single-site phosphorylation for most cold-responsive proteins in cucumber (**Figure 3A**). Additionally, in cucumber, the proportion of Ser, Thr, and Tyr phosphorylation sites was 90%, nearly 10%, and less than 1%, respectively (**Figure 3B**). There was an average of about two Ser phosphorylation sites per protein in total or cold-responsive phosphoproteins and one phosphorylation site per protein in EBR-regulated phosphoproteins. However, Thr and Tyr had only one phosphorylation site, suggesting that multisite phosphorylation occurs in Ser during normal conditions and cold stress (**Figure 3C**).

In this study, all differential phosphopeptides for a protein that are up- or downregulated are called “same-pattern” peptides, and phosphopeptides with opposite regulation for a protein are called “different-pattern” peptides. There were 1,048 cold-responsive phosphopeptides that belonged to the same pattern, corresponding to 675 proteins, accounting for 73.3% of cold-responsive phosphopeptides, and the proportion of same pattern in EBR-regulated cold-responsive peptides and non-cold-responsive peptides was more than 95% (**Figure 3D**; **Supplementary Table S6**), suggesting that phosphorylation of a protein responded to cold or EBR were either up- or downregulated.

We further studied the distribution of up- or downregulated phosphorylation in Ser, Thr, and Tyr. The proportion of upregulated Ser in EBR-regulated cold-responsive phosphopeptides or non-cold-responsive phosphopeptides was significantly higher than that of cold-responsive phosphopeptides (Fisher’s exact test, *p* < 0.005; **Figure 3E**). In addition, the proportion of upregulated phosphopeptides in EBR-regulated cold-responsive phosphopeptides or non-cold-responsive phosphopeptides was also markedly higher than that in cold-responsive phosphopeptides (Fisher’s exact test, *p* < 0.005; **Figure 3F**). These results suggested that EBR regulated cold stress mainly by upregulating Ser phosphorylation of cold- or non-cold-responsive phosphoproteins.

Phosphorylation sites of target proteins provide important information about the activity of the protein kinases involved (Zeng et al., 2014). Phosphorylation motifs were identified from localized phosphorylation sites using the Motif-X (motif extractor) algorithm (Schwartz and Gygi, 2005). We found that only EBR-regulated cold-responsive (EBR-cold) phosphopeptides had significant enrichment motifs as follows. The four types of motifs were xxxRSx_S_xDxxxx, xxxExG_S_xxxxxx, xxxxxx_S_ExDxxx, and xxxxSx_S_Fxxxxx, respectively (**Supplementary Figure S3C**). This is the first identification motifs of the EBR-mediated cold stress response in plants

### 3.4 Hypergeometric analysis of EBR-regulated cold-responsive GO terms and KEGG pathways

**Figure 4:**
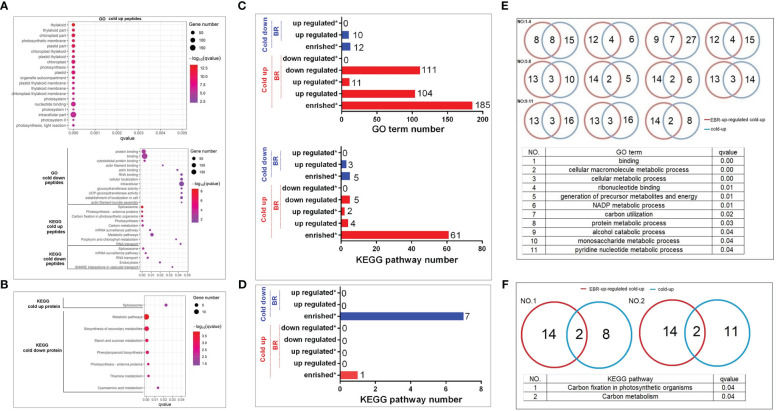
The GO and KEGG enrichment analyses of cold-responsive phosphoproteins and proteins. **(A, C, E, F)** Cold-responsive phosphoproteins; **(B, D)** cold-responsive proteins.

The GO top 20 enrichment analysis found that cold-up phosphoproteins were mainly enriched in photosynthesis and nucleotide binding (**Figure 4A**). The cold-down phosphoproteins were enriched in three categories, including RNA and protein binding, glucosyltransferase activity, and actin filament bundle assembly (**Figure 4A**). KEGG pathway enrichment analysis revealed that cold-up phosphoproteins enriched in four categories, including spliceosome, photosynthesis, RNA transport and surveillance, carbon metabolism; and cold-down phosphoproteins enriched in four categories, including spliceosome, RNA transport and surveillance, endocytosis, and vesicular transport (**Figure 4A**). As for differential proteins in the proteome, the KEGG pathway enrichment analysis revealed that only the spliceosome pathway was enriched in cold-up proteins. The cold-down proteins were enriched in five categories, including starch and sucrose metabolism, phenylpropanoid biosynthesis, photosynthesis antenna proteins, thiamine metabolism, and cyanoamino acid metabolism (**Figure 4B**; **Supplementary Table S7**).

We investigated the importance of EBR in the regulation of cold-responsive phosphoproteins and proteins by using a hypergeometric test (**Supplementary Table S8**). However, different from the EBR regulation at the omics level, the cold-up phosphoproteins enriched 185 GO terms, among which 104 and 111 GO terms were respectively up- or downregulated by EBR, and 11 GO terms were significantly upregulated by EBR (**Figure 4C**), including molecular binding, energy metabolism, alcohol and glucose metabolism, etc. For example, there were 24 cold-up phosphoproteins involved in binding GO terms, among which eight were upregulated by EBR (**Figure 4E**; **Supplementary Table S8**). Cold-down phosphoproteins only enriched 12 GO terms, which were significantly lower than the proportion of cold-up GO terms (proportion test, *p* < 0.01), among which 10 GO terms were downregulated by EBR but no one was enriched (**Figure 4C**). Further analysis of the KEGG pathway showed that cold-up phosphoprotein enriched 61 pathways, among which four and five pathways were respectively up or downregulated by EBR, and two pathways were significantly upregulated by EBR (**Figure 4C**), including carbon fixation of photosynthesis and carbon metabolism (**Figure 4F**). For example, there were 10 cold-up phosphoproteins involved in carbon fixation in the photosynthesis pathway, among which two phosphoproteins were regulated by EBR (**Figure 4C**). While in the proteome, cold-responsive proteins did not enrich for GO terms, and there were eight KEGG pathways enriched in cold-up and cold-down proteins (**Figure 4D**). Nevertheless, no one was regulated by EBR.

The above analysis suggested that EBR regulated the GO terms and KEGG pathways in response to cold stress mainly through upregulation of nucleotide binding, energy metabolism, and phosphorylation levels of cold-up phosphoproteins. Therefore, EBR regulated cold stress response mainly through upregulating the 16 cold-up phosphoproteins in cucumber.

### 3.5 Construction of cold-responsive protein and phosphoprotein interaction networks and functional analysis of interaction proteins

**Figure 5:**
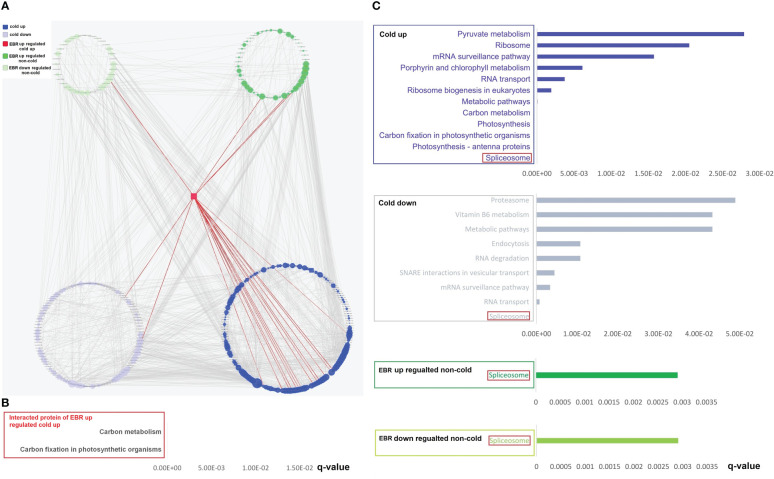
The PPI networks’ construction and KEGG pathway analysis for differential phosphoproteins. **(A)** The construction of PPI networks for differential phosphoproteins; KEGG pathway of interacted proteins of EBR-upregulated cold-up **(B)**, cold-up, cold-down, EBR-upregulated non-cold, and EBR-downregulated non-cold phosphoproteins **(C)**.

The String 15.0 database was further used for the analysis of the interaction of differential phosphoproteins and proteins, and then the PPI network was constructed by Cytoscape software. We first constructed the PPI network of differential phosphoproteins (**Supplementary Table S9**). The complex network consisted of 352 nodes and 2,635 edges (**Supplementary Table S9**), with five categories, including 161 cold-up, 109 cold-down, one EBR-upregulated cold-up, 47 EBR-regulated non-cold-up, and 34 EBR-downregulated non-cold. The percentage of cold-up phosphoproteins in total interaction phosphoproteins was significantly higher than others (proportion test, *p* < 0.01). The number of phosphoproteins that interacted with cold-up phosphoproteins was 1,389, cold-down was 675, EBR-upregulated cold-up was 21, EBR-upregulated non-cold was 296, and EBR-downregulated non-cold was 254. The percentage of interaction times with cold-up phosphoproteins in total interaction times was significantly higher than that with other phosphoproteins categories (proportion test, *p* < 0.01). These results revealed that the cold-up phosphoproteins may have played a dominant role in protein interactions under cold stress. Hypergeometric test analysis had shown that EBR was primarily involved in cold-up phosphoproteins (**Figure 5A**). We also obtained one protein kinase protein (Csa6G136020) belonging to the EBR-upregulated cold-up category through PPI (**Figure 5A**). This phosphoprotein interacted with 15 cold-up phosphoproteins, two cold-down phosphoproteins, three EBR-upregulated non-cold phosphoproteins, and one EBR-downregulated non-cold phosphoprotein, and these proteins were involved in carbon fixation in photosynthetic organisms and carbon metabolism through KEGG enrichment analysis (**Figures 5A, B**; **Supplementary Table S9**). Therefore, at the protein interaction level, EBR might regulate carbon fixation in photosynthetic organisms and carbon metabolism pathways through upregulating phosphorylation of Csa6G136020.

In order to further clarify the functions of the four categories of interacting proteins (except for the EBR upregulated cold-up), KEGG enrichment analysis was conducted for them, and the results showed that the spliceosome was the most significantly enriched pathway for the four categories of interacting proteins. These results suggest that cold-responsive phosphoproteins might interact with EBR-regulated non-cold phosphoproteins to regulate spliceosomes, responding to cold stress in cucumbers (**Figures 5C**).

Additionally, there are only 32 differential proteins for the PPI network in the proteome, among which the number of cold-down proteins was 14, whose percentage of total interacting proteins was significantly higher than other categories of proteins (proportion test, *p* < 0.01). These results suggest that cold-down protein may significantly regulate protein interaction in cucumbers under cold stress. The 32 proteins formed a total of 10 protein interaction networks (**Supplementary Figure S4**), and the percentage of interacting proteins in the first category was significantly higher than that of other proteins (proportion test, *p* < 0.001). However, there was no KEGG pathway enriched in this category.

### 3.6 Analysis of the relationship between phosphorylation levels and protein contents

**Figure 6:**
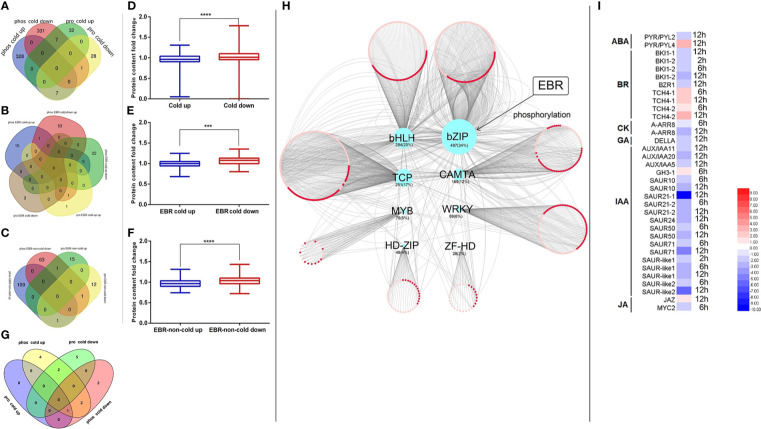
The association analysis of the transcriptome, proteome, and phosphoproteome. **(A)** The Venn of cold-responsive phosphopeptides and cold-responsive proteins; **(B)** the Venn of EBR-regulated cold-responsive phosphopeptides and EBR-regulated cold-responsive proteins; **(C)** the Venn of EBR-regulated non-cold-responsive phosphopeptides and EBR-regulated non-cold-responsive proteins; **(D)** the cold-responsive protein content between cold-up and cold-down phosphoproteins; **(E)** the EBR-regulated cold-responsive protein content between EBR-upregulated cold and EBR-downregulated cold-responsive phosphoproteins; **(F)** the EBR-regulated non-cold protein content between EBR-upregulated non-cold and EBR-downregulated non-cold phosphoproteins; **(G)** the Venn of KEGG pathway between differential phosphoproteins and proteins; **(H)** the network construction of cold TFs and its corresponding cold target mRNA; and **(I)** the expression of hormone signaling pathway genes under cold stress. The "***" indicates significantly different (p < 0.001 by Tukey’s test), and "****" indicates significantly different (p < 0.0001 by Tukey’s test).

Transcriptome and proteome association analysis found only 10 differential common genes between them (**Supplementary Figure S5**), indicating that most of the differentially expressed mRNAs did not cause protein differences, which may be due to the effect of post-translational regulatory mechanisms on protein content. To investigate the association between protein phosphorylation signal and protein content, we examined the connection between cold-responsive phosphoproteins of phosphoproteome and cold-responsive proteins in the proteome (**Figure 6A**). We discovered there were seven common proteins in phos_cold down (phos represents phosphorylated) and pro_cold up (pro represents protein), seven common proteins between phos_cold up and pro_cold down, one consensus protein between phos_cold down and pro_cold down (**Figure 6A**). Alternatively, for EBR-regulated proteins, there was one consensus protein between phos_EBR cold-down up and pro_EBR cold-up up (**Figure 6B**), one consensus protein between phos_EBR-non-cold down and pro_EBR-non-cold up (**Figure 6C**), one consensus protein between phos_EBR-non-cold-up and pro_EBR-non-cold down, and one consensus protein between phos_EBR-non-cold-down and pro_EBR-non-cold down (**Figure 6C**). To summarize, we found that the common proteins of the phosphoproteome and proteome included 15 cold-responsive proteins (**Figure 6A**), one EBR-regulated protein (**Figure 6B**), and three EBR-regulated non-cold-responsive proteins (**Figure 6C**). There were 19 proteins in total, among which the number of proteins with a negative correlation between phosphorylation signal and protein content was 16, accounting for 84.21% of all common proteins (proportion test, *p* < 0.01).

By associating the KEGG pathway of differentially expressed proteins in the proteome and phosphoproteome, only chilling-responsive proteins had an overlap of the pathway (**Figure 6G**). In phos_cold up and pro_cold down, two co-pathways showed a negative correlation: “photosynthesis—antenna proteins (csv00196)” and “metabolic pathways (csv01100)” (**Figure 6G**).

Furthermore, the relationship between phosphorylated up-regulated proteins and phosphorylated downregulated proteins was further analyzed from the perspective of omics (**Figures 6D–F**). We discovered that the content of phosphorylated downregulated proteins was significantly higher than that of phosphorylated upregulated proteins (**Figure 6D**). Nevertheless, the content of phosphorylated downregulated proteins was significantly higher than that of phosphorylated upregulated proteins regulated by EBR (**Figures 6E, F**). In conclusion, protein content was negatively correlated with its phosphorylation level in cucumber.

### 3.7 The multi-omics analysis of cold regulation transcription factors and their target genes

In order to investigate whether EBR plays an important role in cold-responsive transcription factor (TF) regulation, we extracted differential cold-responsive TFs from the phosphoproteome and proteome and then obtained the corresponding transcriptome of TFs. We found that differential TFs mainly focused on the phosphoproteome, but these TFs were not differential mRNA and protein (**Supplementary Figure S6**), suggesting that the activity of TFs may be mainly regulated by phosphorylation signals under cold stress. TF target genes were further screened in the transcriptome (**Supplementary Table S10**), and then the network was constructed with Cytoscape (**Figure 6H**). A total of eight types of differentially phosphorylated TFs were regulated by cold stress, among which the target genes of bZIP TFs were the most, accounting for 34% of the total number of target genes (**Figure 6H**).

To further study the potential function of TFs in response to cold stress, we conducted the KEGG pathway enrichment analysis on target genes of different types of TFs. It was found that target genes regulated by bZIP (including CsABI5.2 and CsABI5.5) were significantly enriched, mainly participating in plant hormone signal transduction (**Supplementary Figure S7**), and most genes related to auxin (IAA), cytokinin (CK), and gibberellin (GA) pathways were significantly downregulated under cold stress (**Figure 6I**), while some genes related to BR, jasmonic acid (JA), and abscisic acid (ABA) pathways were upregulated (**Figure 6I**). Notably, the phosphorylation levels of these two TFs were downregulated under cold stress; however, EBR was involved in regulating hormone signal transduction by upregulating CsABI5.2 at S318 and CsABI5.5 at S293 (**Figure 6I**; **Supplementary Table S2**).

**Figure 7:**
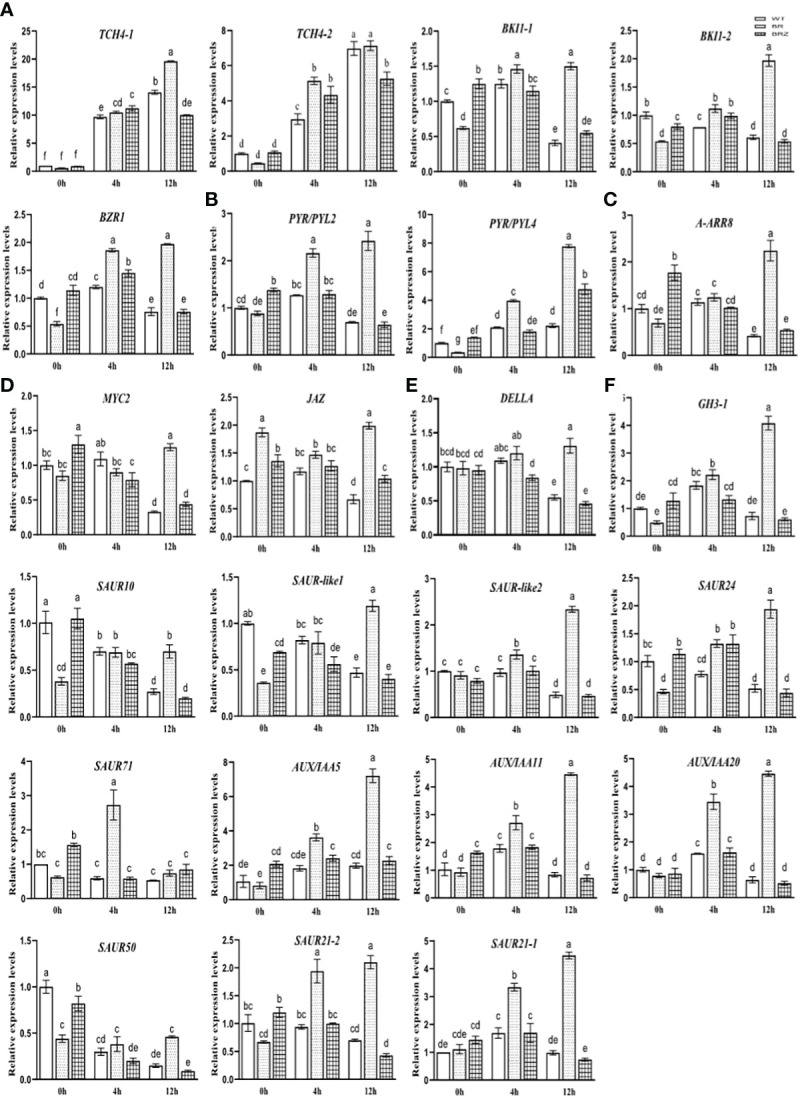
The expression of hormone signaling pathway genes, BR **(A)**, ABA **(B)**, CK **(C)**, GA **(D)**, IAA **(E)**, and JA **(F)**, regulated by EBR under cold stress. Data are mean ± SE (*n* = 3, biological replicates). The different letters were significantly different in Tukey’s test (*p* < 0.05).

The expression of these hormone-related genes (**Figure 6I**) was then verified, and we found that the gene expression determined by qRT-PCR in cucumber tended to be consistent (the consistent rate was 82.61%), indicating that the sequencing results had high accuracy and reliability (**Figures S7A–F**). However, studies on EBR that were involved in regulating various hormone signaling pathways under cold have not been reported. In this study, EBR treatment significantly enhanced the expression of BR, ABA, and IAA (66.67%; except *CsGH3-1*, *CsSAUR10*, *CsSAUR-like1*, and *CsSAUR50*) genes compared with wild type at 4 h for 4°C and the expression of BR (80%, except *CsTCH4-2*), ABA, JA, CK, GA, and IAA (91.67%, except *CsSAUR71*) genes compared with wild type at 12 h for 4°C. These results suggested that EBR treatment promoted these genes’ expression, including abiotic stress and growth hormone genes. However, BRZ treatment suppressed the expression of BR (40.00%; *CsBKI1-1*, *CsBZR*), ABA, GA, and IAA (83.33%; except *CsSAUR10* and *CsSAUR24*) genes compare with EBR treatment at 4 h for 4°C, and the expression of BR, ABA, JA, CK, GA, and IAA (91.67%, except *CsSAUR71*) genes compared with EBR treatment at 12 h for 4°C (**Figures 7A–F**).

## 4 Discussion

As the basic substance of life, proteins play important roles in life activities. Environmental conditions affect the stability and activity of proteins. In order to understand how the plant responds to different environments, it is crucial to analyze changes in the proteome (Nelofer et al., 2019) and PTMomes, including phosphoproteome (Kosová et al., 2021). The cold stress response in cucumbers is a complex process involving a large number of genes and proteins. Although some cold stress tolerance genes have been identified in plants, multiple omics analyses of the transcriptome, proteome, and phosphorylation for plants under cold stress have still not been reported. Furthermore, it is well known that BR plays an essential role in cold signal integration, regulating downstream cold-responsive genes (Eremina et al., 2016b; Li et al., 2017; Ye et al., 2019), and spraying EBR significantly enhanced the cold tolerance of plants (Xia et al., 2014; Anwar et al., 2018; Bai et al., 2018; Dou et al., 2021). However, in plants, how EBR regulates the phosphoproteome and proteome under cold stress has still not been reported. Here, multi-omics revealed that cucumber responded to cold stress mainly *via* protein phosphorylation, and EBR further reprogrammed the phosphorylation of cold phosphoproteins, affecting its cold response.

### 4.1 EBR induced single-site phosphorylation under cold stress and mainly upregulated Ser phosphorylation in response to cold stress

In this study, 90% of the phosphorylated amino acid were Ser, followed by Thr and Tyr (**Figure 3B**), which was in agreement with studies in bananas (Gao et al., 2017), *Arabidopsis* (Wang et al., 2019a; Gao et al., 2021), tomato (Hsu et al., 2018), Mulberry (Pi et al., 2017), and cotton (Ma et al., 2014), suggesting that the protein phosphorylation of plant primarily occurred in the Ser residues. This study also showed that cucumber had an average of two phosphorylation sites per cold-responsive protein (**Figure 3A**), while *Arabidopsis* and tomato have an average of only one site per cold-responsive protein (Gao et al., 2017; Hsu et al., 2018; Kamal et al., 2020). Compared with other crops, cold regulation phosphorylation sites were more abundant in cucumber, further indicating that cucumber was modified mainly by phosphorylation in response to cold. The phosphoproteins of EBR-regulated cold and EBR-regulated non-cold had only one phosphorylation site (**Figure 3A**), indicating the specificity of EBR in the regulation of protein phosphorylation. We also found that multiple phosphorylation sites for a protein were consistent in response to cold stress or EBR, either up- or downregulated (**Figure 3D**). Furthermore, unlike cucumber, the number of downregulated cold-responsive phosphopeptides in banana and *Arabidopsis* was far more than upregulation cold-responsive phosphopeptides. For example, 48 cold-upregulation and 132 cold-downregulation phosphopeptides have been identified in bananas (Gao et al., 2017); there were 707 cold-upregulation and 1,334 cold-downregulation phosphopeptides identified in *Arabidopsis* (Gao et al., 2021). These results illustrated that the phosphorylation modification of cold-responsive phosphopeptides was different in diverse species. Notably, the proportion of upregulated Ser in EBR-regulated cold-responsive phosphopeptides or non-cold phosphopeptides was significantly higher than those in cold-responsive phosphopeptides (**Figure 3E**), suggesting that EBR responded to cold stress mainly by upregulating Ser phosphorylation of cold or non-cold phosphopeptides. The four types of motifs were: xxxRSx_S_xDxxxx, xxxExG_S_xxxxxx, xxxxxx_S_ExDxxx, and xxxxSx_S_Fxxxxx, respectively (**Supplementary Figure S3C**). This is the first identification motif of the EBR-mediated cold stress response in plants.

### 4.2 EBR regulated cold-responsiveness by regulating phosphorylated proteins related to photosynthetic, nucleotide bindings, and spliceosome

EBR affects abiotic stress resistance in plants (Kohli et al., 2018; Xia et al., 2018; Zhou et al., 2018; Wang et al., 2019b; Li et al., 2021), whereas how EBR regulates the cold-responsive of plants in phosphoproteome and proteome has not been reported. We first investigated the effect of EBR on phosphoproteome and proteome. Regardless of the phosphoproteome or proteome, EBR reprogrammed the protein response to cold stress through negatively regulated phosphorylation levels of cold-responsive phosphopeptides and the content of cold-responsive proteins in cucumber seedlings (**Figures 2A–D**).

Further enrichment analysis of differential genes revealed that cucumber mainly responded to cold stress *via* nucleotide binding and photosynthesis in cold-up phosphoproteins and sucrose metabolism in proteins (**Figures 4A, B**). EBR negatively regulates cold-responsive phosphopeptides in the proteome; however, the hypergeometric test shows that EBR is involved in 56.22% GO terms and 6.56% of KEGG pathways; 11 GO terms (photosynthesis and nucleotide binding) and two pathways (photosynthesis) were significantly upregulated by further unregulated 16 cold-up phosphoproteins (**Figures 4C, D**). These results suggested that EBR affected photosynthesis and nucleotide binding by upregulating 16 cold-up phosphoproteins of cucumber (**Figures 4E, F**). Of these, fructose-1,6-bisphosphate aldolase (FBA, Csa_2G252020)was proved to be positively correlated with cold tolerance in tomatoes (Cai et al., 2017), and it was significantly expressed as a cold-responsive protein in *Arabidopsis thaliana* (Li et al., 2014). The CML family protein (Csa_1G025020) was detected in transcript profiles of chrysanthemum leaves exposed to −8°C (Lu et al., 2018), and the villin-4 (Csa_2G060390) can respond to low temperature in cotton (Lv et al., 2021).

Furthermore, in PPI, EBR affected cold-responsive phosphoproteins through regulated non-cold phosphoproteins in the spliceosome and regulated photosynthesis through regulated cold-up phosphoproteins. In general, EBR may regulate photosynthesis, spliceosomes, and nucleotide binding by regulated non-cold and cold phosphoproteins (**Figure 5**). Similar findings were seen with the other plants. The PPI of maize root under low temperatures was significantly enriched in functions of biosynthetic processes and photosynthesis (Ma et al., 2019). In rice, the PPI was also an enrichment in photosynthesis (Sperotto et al., 2017).

### 4.3 The association’s analysis of the proteome and phosphoproteome reveals that phosphorylation negatively regulates the proteome in cucumber

Previous studies found that the phosphorylation of cold-responsive proteins in bananas had a low correlation with protein content (Gao et al., 2017). Protein phosphorylation modification in Arabidopsis also changed significantly after cold stress, but no changes were observed in protein content levels (Gao et al., 2021). However, in cucumber, the study found that the common differential proteins of the phosphoproteome and proteome included 15 cold-responsive proteins (**Figure 6A**), one EBR-regulated protein (**Figure 6B**), and three EBR-regulated non-cold-responsive proteins (**Figure 6C**), among which the number of proteins with a negative correlation between phosphorylation signal and protein content was 16, accounting for 84.21% of all common proteins. Furthermore, the content of downregulated phosphopeptides was significantly higher than that of upregulated phosphopeptides (**Figures 6D–F**). Moreover, there were three common pathways in the phosphoproteome and proteome, among which the phosphorylated signals of two pathways were negatively correlated with protein content, including photosynthesis-antenna proteins and metabolic pathways (**Figure 6G**). In general, the above results strongly indicated that the active phosphorylation of protein in cucumber was not conducive to increasing protein content under cold stress. Furthermore, we also found that EBR downregulated cold-up phosphopeptides (**Figure 3C**) and upregulated cold-down phosphopeptides in phosphoproteome (**Figure 3D**).

### 4.4 Multi-omics analysis reveals that EBR enhanced the expression of hormone-signaling genes, which was linked to phosphorylation

TFs are crucial in controlling gene expression (Huang et al., 2018). The above study found that cucumber responded to low temperature through nucleotide-binding-related protein phosphorylation, which was significantly regulated by EBR (**Figure 4**). Therefore, further analysis of transcription factor regulation was required. We found that differential TFs mainly focused on phosphoproteome, but these TFs were not differential mRNA and protein (**Supplementary Figure S6**), suggesting that the activity of TFs was mainly regulated by phosphorylation signal under cold stress. There were eight types of TFs in response to cold stress in cucumbers, including bHLH, bZIP, TCP, MYB, WRKY, CAMTA, HD-Zip, and ZF-H (**Figure 6H**). However, *Arabidopsis* only has three TFs regulated by cold stress, including C3H, bZIP, and CAMTA (Gao et al., 2021). The bZIP family, as one of the largest and most diverse TF families, is an important participator in abiotic stress responses and hormone signaling (Wang et al., 2015; Dröge-Laser et al., 2018). In this study, we also found that only target genes for bZIP TFs (CsABI5.2 and CsABI5.5) were significantly enriched and participated in hormone signaling transduction (**Figure 6**; **Supplementary Figure S7**). Moreover, bZIP TFs were further regulated by EBR (**Figure 6**).

We then explored how EBR affected cold-related hormone gene expression. In this study, we found that some genes related to the BR, JA, and ABA pathways were upregulated, but most genes related to IAA, CK, and GA pathways were significantly downregulated, suggesting that the growth of cucumber seedlings was inhibited under cold stress (Verma et al., 2016; Eremina et al., 2016a) (**Figure 6I**). However, EBR strongly increased most of the expression of stress-related (JA, ABA, and BR) and growth-related hormone (IAA, GA, and BR) genes compared with the wild type under cold stress, suggesting EBR might simultaneously promote cold stress resistance and growth of cucumber seedlings (**Figure 7**). While the previous study also showed that in cold-sensitive tomatoes, cold stress resulted in the decrease in the content of GA and IAA, while EBR robustly increased the content of GA, IAA, and ABA (Heidari et al., 2021), showing consistently in this study. In general, we first indicated that EBR could phosphorylate CsABI5.5 and CsABI5.2, thus changing the response to cold stress by upregulating the most of the hormone signal gene expression in cucumber.

### 4.5 EBR-mediated schematic of response mechanisms in cucumber under cold stress

**Figure 8:**
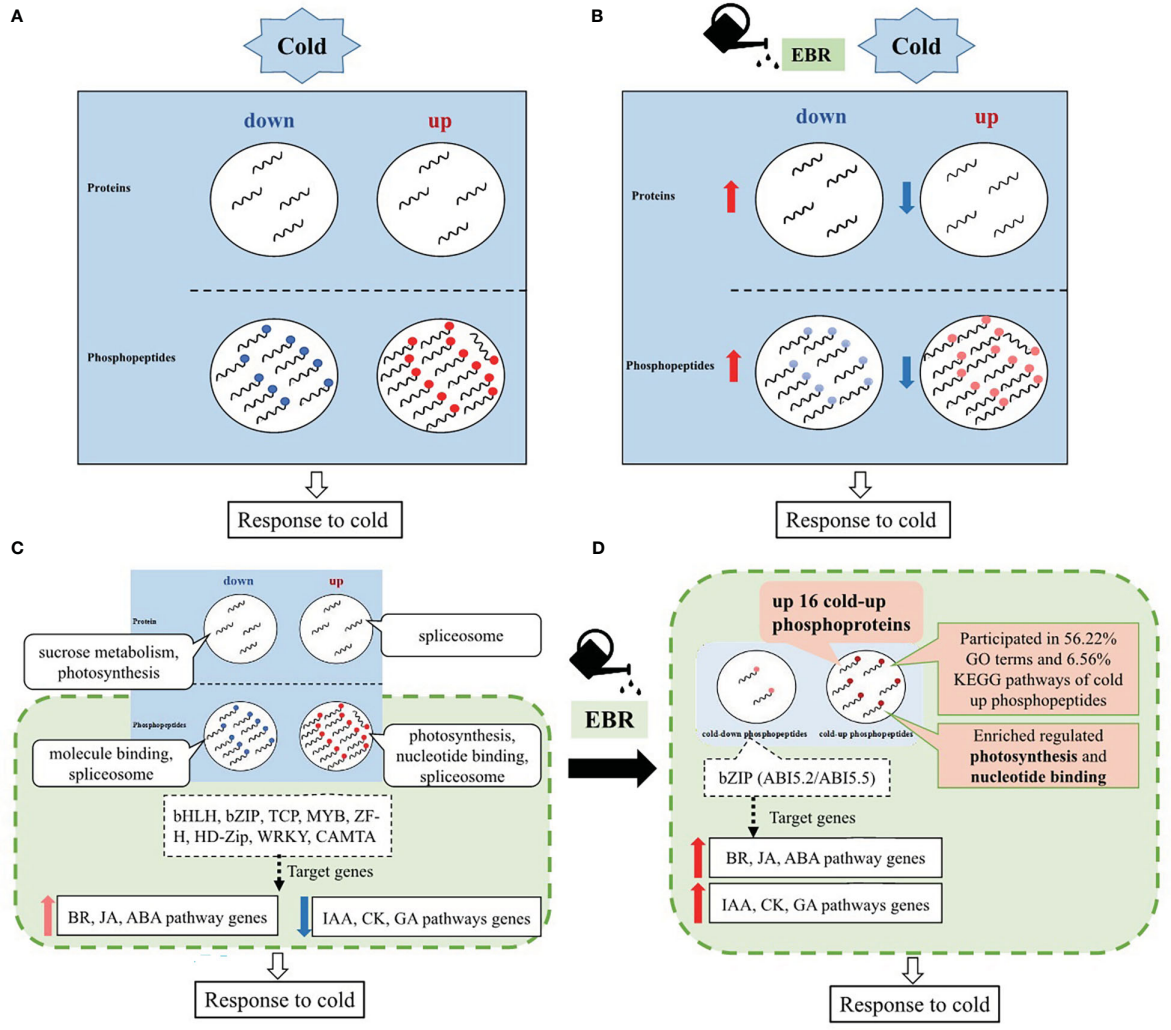
Schematic representation of a possible EBR-regulated comprehensive cold response model in cucumber. **(A)** The effects of cold stress on proteins and phosphopeptides of cucumber; **(B)** the effects of EBR on cold-responsive proteins and phosphopeptides of cucumber; **(C)** a schematic representation of a comprehensive cold response model of cucumber; and **(D)** a schematic representation of EBR-regulated comprehensive cold response model of cucumber.

Based on the above results, an EBR-mediated systematic cold response in cucumber was proposed (**Figure 8**). Cucumber responded to cold stress mainly through protein phosphorylation (**Figure 8A**), while EBR reprogrammed the proteins’ response to cold stress by negatively regulating cold-responsive phosphopeptides and proteins (**Figure 8B**). That is, EBR downregulated cold-up phosphopeptides but upregulated cold-down phosphopeptides. Similarly, EBR downregulated cold-up proteins but upregulated cold-down proteins (**Figure 8B**). These results suggested that most phosphopeptides and proteins were negatively regulated by EBR; however, the hypergeometric analysis showed that positive regulation by EBR was the key response mechanism by upregulating 16 cold-up phosphoproteins in cucumber seedlings.

Further analysis on enrichment differential proteins and phosphopeptides showed that EBR regulated cold stress *via* phosphorylation with upregulated 16 cold-up phosphoproteins participating in photosynthesis and nucleotide binding, which occupied 56.22% GO terms and 6.56% KEGG pathways for cold-up phosphopeptides in total (**Figure 8D**). Moreover, cucumber regulated TFs also through protein phosphorylation that included eight classes of TFs, and only bZIP target genes were enrichment in hormone signal genes in response to cold stress. Most hormone genes related to growth (IAA, CK, and GA) were significantly downregulated, and those related to abiotic stress (BR, JA, and ABA) were upregulated (**Figure 8C**), while EBR further increased the bZIP phosphorylation level and hormone signaling gene expression (**Figure 8D**).

## 5 Conclusion

Phosphoproteome analysis showed that cucumber responded to cold stress through multi-site serine phosphorylation, while EBR further upregulated single-site phosphorylation for most cold-responsive phosphoproteins. Association analysis of the proteome and phosphoproteome revealed that EBR reprogrammed proteins in response to cold stress by negatively regulating protein phosphorylation and protein content, and phosphorylation negatively regulated protein content in cucumber. Furthermore, protein interaction networks and functional enrichment analysis of the proteome and phosphoproteome showed that cucumber mainly upregulated phosphoproteins related to spliceosomes and photosynthetic pathways in response to cold stress. Moreover, the hypergeometric analysis showed that EBR further upregulated 16 cold-up-responsive phosphoproteins that participated in photosynthetic and nucleotide-binding pathways in response to cold stress, suggesting their important function in cold tolerance. Analysis of cold-responsive transcription factors (TFs) by the correlation between proteome and phosphoproteome showed that cucumber-regulated eight-class TFs may be through protein phosphorylation. Further analysis of the cold-related transcriptome found that cucumber phosphorylated eight class TFs targeted five major hormone signal genes in response to cold stress, while EBR further increased the bZIP TFs’ (CsABI5.2 and CsABI5.5) phosphorylation levels and hormone signaling genes’ expression.

## Data availability statement

The data presented in the study are deposited in the iProX partner repository, accession number PXD040072.

## Author contributions

MS and XY conceived and designed the study. MZ performed the experiments. MZ collected the data. MS and MZ performed mapping and bioinformatics analysis. MZ and MS prepared the manuscript. MS and XY provided guidance on the whole study. All authors contributed to the article and approved the submitted version.
