# Supplementary figures and images for: Proteome and phosphoproteome analysis of 2,4-epibrassinolide-mediated cold stress response in cucumber seedlings
Source: Front Plant Sci. 2023 Feb 21;14:1104036. doi: 10.3389/fpls.2023.1104036 (PMC9989176; doi:10.3389/fpls.2023.1104036)

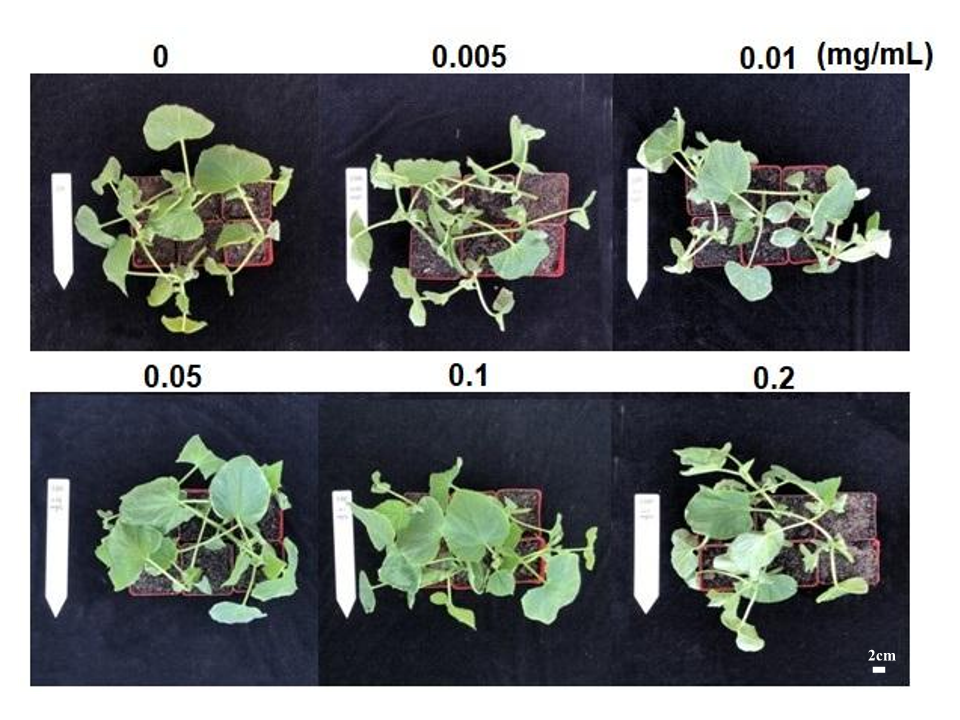

Supplement: Supplementary file 1 [file Image_1.jpg]

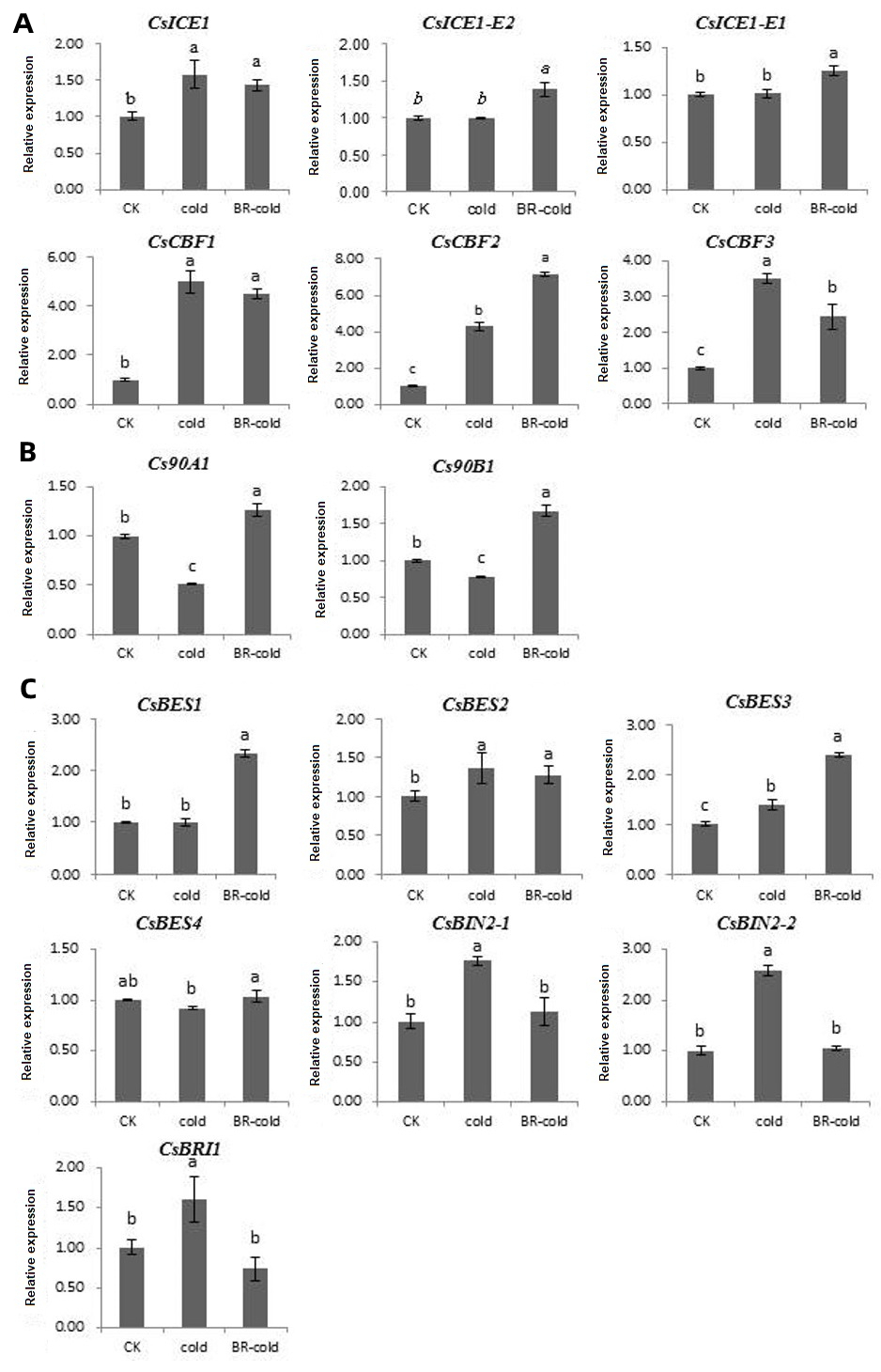

Supplement: Supplementary file 2 [file Image_2.jpg]

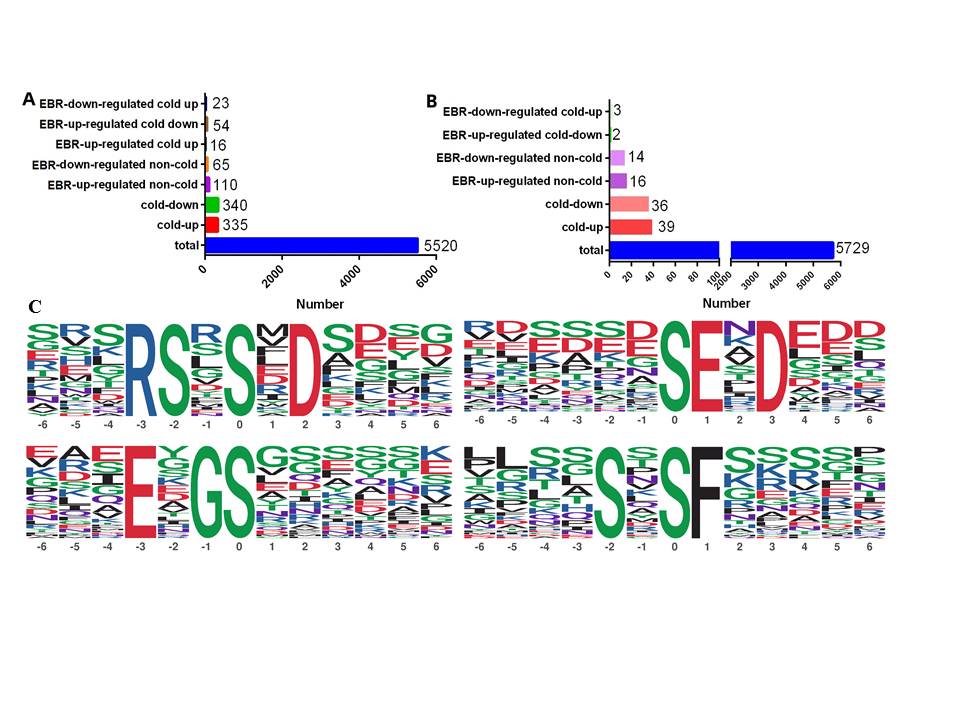

Supplement: Supplementary file 3 [file Image_3.jpg]

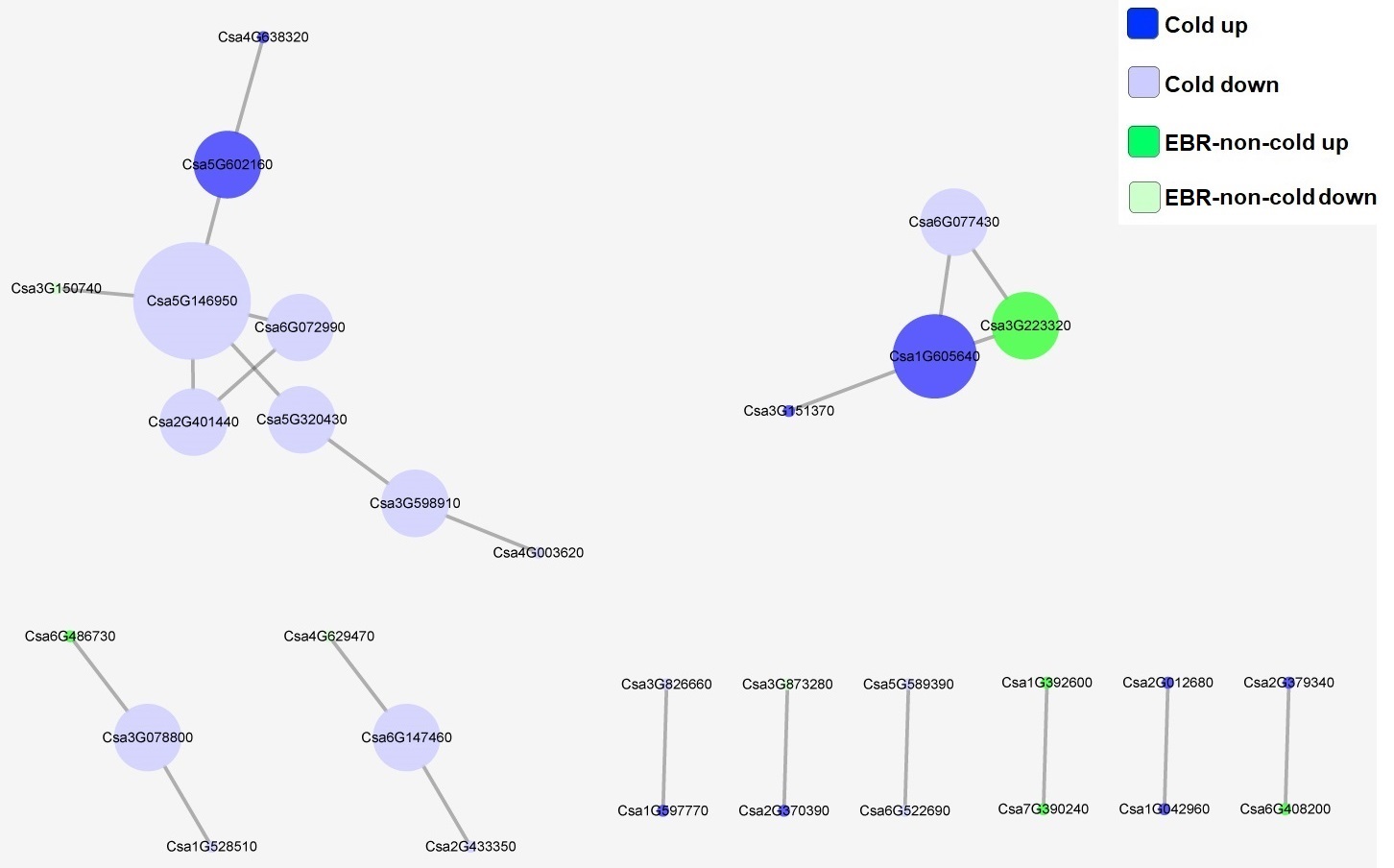

Supplement: Supplementary file 4 [file Image_4.jpg]

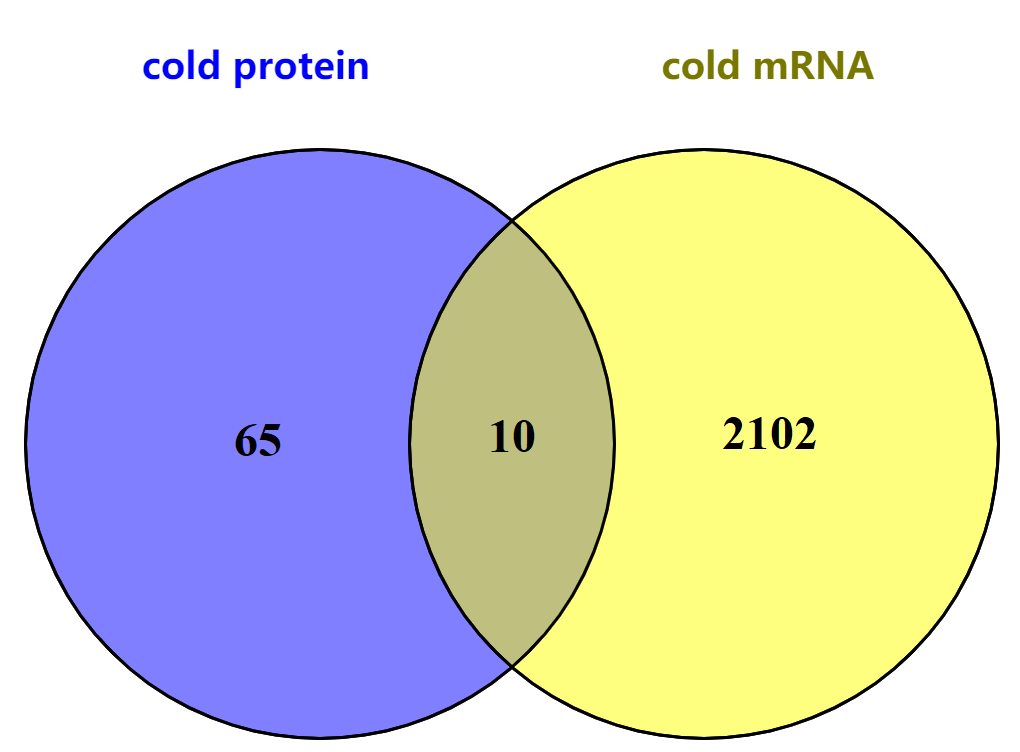

Supplement: Supplementary file 5 [file Image_5.jpg]

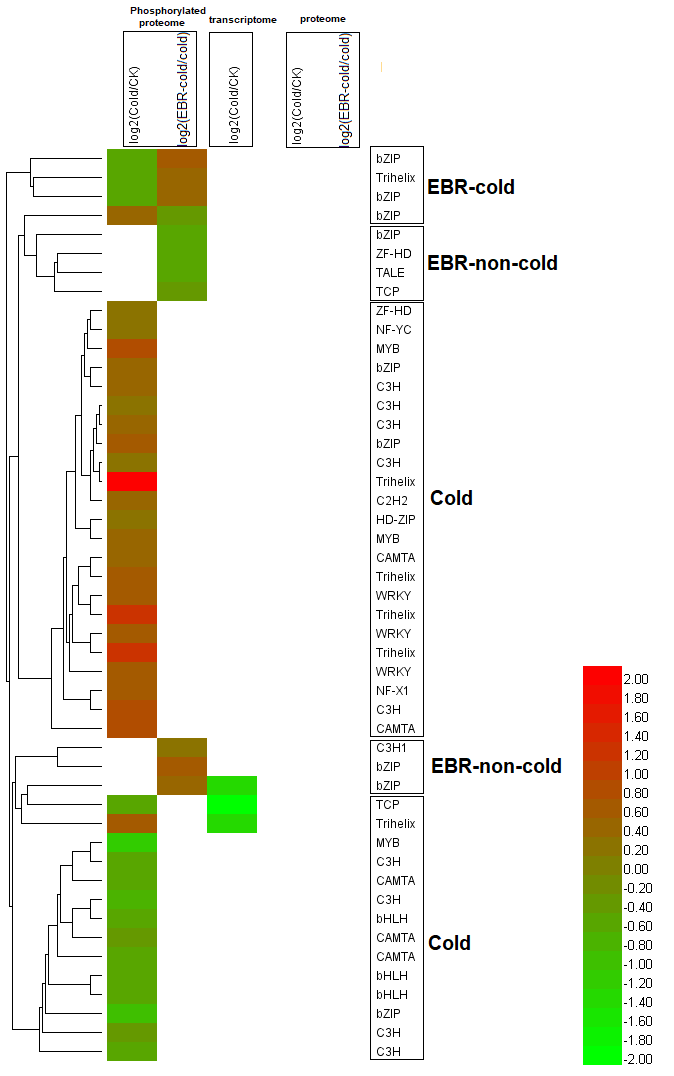

Supplement: Supplementary file 6 [file Image_6.jpg]

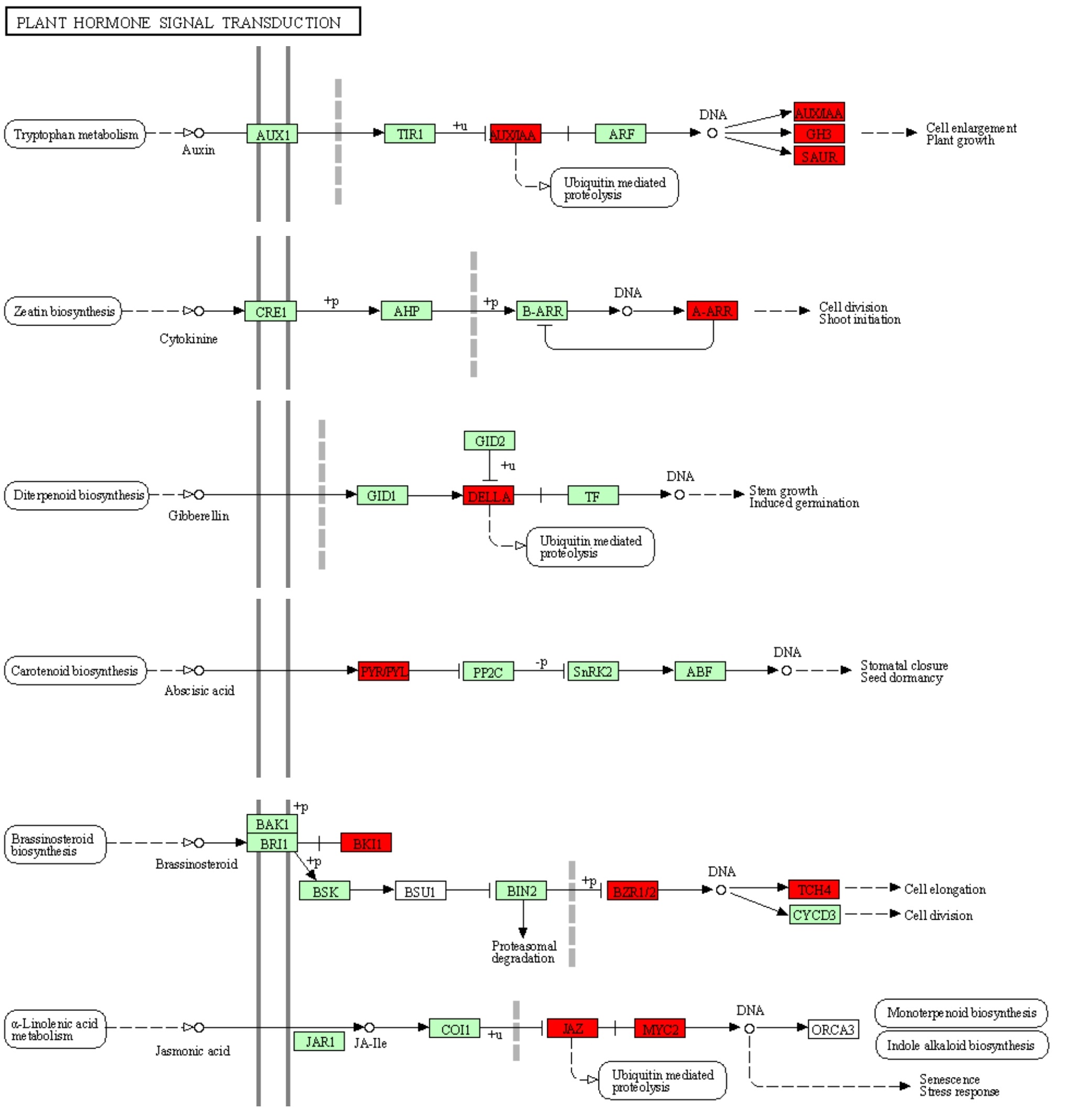

Supplement: Supplementary file 7 [file Image_7.jpg]
